# Case Report: Acute Onset Fear of Falling and Treatment With “Cognitive Physical Therapy”

**DOI:** 10.3389/fneur.2021.707840

**Published:** 2021-08-06

**Authors:** Patricia Castro, Shree Vadera, Matthew James Bancroft, Joseph Buttell, Diego Kaski

**Affiliations:** ^1^Centre for Vestibular and Behavioural Neurosciences, Department of Clinical and Movement Neurosciences, University College London, London, United Kingdom; ^2^Department of Neuro-Otology, Division of Brain Sciences, Imperial College London, Charing Cross Hospital, London, United Kingdom; ^3^Universidad del Desarrollo, Escuela de Fonoaudiología, Facultad de Medicina Clínica Alemana, Santiago, Chile; ^4^Regional Neurological Rehabilitation Unit, Homerton University Hospital Foundation National Health Service Trust, London, United Kingdom

**Keywords:** fear of falling, falls, postural anxiety, cognitive behavioural therapy, cognitive physical therapy

## Abstract

Fear of falling (FoF) is prevalent in older adults, especially those with previous falls, and typically starts insidiously. We present a 78-year-old woman with an abrupt onset FoF and no history of falls, balance problems, vertigo, oscillopsia, psychiatric or psychological issues to account for this. These cognitive changes led to a behavioural alteration of her gait that became slow and wide-based, with her gaze fixed on the floor. She began a tailored program of “Cognitive Physical Therapy (CPT)” combining cognitive behavioural therapy (CBT) and physical rehabilitation. 1 month later her 6 m walk time and steps were reduced by a 25 and 35%, respectively, and the stride length increased by 34%, with further improvement 2 months later. We postulate that the abrupt onset of symptoms triggered a central shift toward postural hypervigilance and anxiety, suppression of anticipatory (feed forward) postural adjustments (APA) leading to FoF. CPT improved objective gait parameters related to FoF and reduced postural anxiety suggesting that early diagnosis and prompt treatment may avoid chronic symptoms and social isolation.

## Introduction

Fear of falling (FoF) is an abnormal psychological or cognitive response to one's perception of stability, usually developing insidiously. It has a high prevalence, especially in community-dwelling older adults (1), and often leads to behavioural changes in walking patterns. Whilst a cause is not always obvious, the debilitating fear leads to a vicious cycle of avoidance of physical activity (2) and progressive physical disability (1), which in itself may be a precursor to actual falls. Sufferers of FoF may also modify their gait in detrimental ways, including a slower gait and a reduced stride length, which can further increase their risk of falling (3). Limited data exists regarding the neural pathways involved in FoF, and studies have instead focussed on the biomechanical or environmental multifactorial nature of the condition.

Several treatment interventions have been trialled in patients with FoF with varying success. These include integrated exposure therapy (4), cognitive behavioural therapy (CBT) (5) and exercise training (6). However, most interventions have focussed on either the biomechanical manifestations that underpin poor balance (strength, range of motion, endurance, balance skills), or primarily on the psychological fear responses that often lead to task avoidance strategies (7).

Here we present the case of a 78-year-old woman with an abrupt onset FoF that improved through targeted cognitive physical therapies. We discuss the interaction between brain mechanisms and cognitive function in FoF and describe an effective intervention to tackle this condition.

## Case Description

A 78-year-old Caucasian woman presented at age 74 with a sudden sensation of imbalance whilst walking down a sloping road and feeling herself being propelled forwards. She immediately held onto a passer-by to keep her balance. Since then, she has suffered a persistent sensation of imbalance and a constant fear of falling if unsupported, which led her to the immediate avoidance of daily life activities such as walking unaided, despite no physical impediment. Before this episode, the patient led an active life, without any limitations to her daily activities. She had no history of falls, balance problems, vertigo, oscillopsia, or hearing loss, either reported at consultation or on her medical records. She reported no sensory symptoms and had no relevant neurological history of note. She had been taking alendronate, vitamin D, atorvastatin, levothyroxine, ramapril and indapamide for several years with no recent change to the doses. There was no prior history or record of any psychiatric or psychological issues or mental illness to account for the balance disorder. She lives with her husband and reported good family relationships and strong social support networks. She scored 1 on the Clinical Frailty Scale, equivalent to “very fit” (8) for her age. On examination her gait was slow and wide-based, and her gaze remained fixed on the floor (Supplementary File 1). Ankle reflexes were absent bilaterally, but the remainder of the neurological examination was normal. She had normal corrected visual acuities, no spontaneous or gaze-evoked nystagmus, normal positional (Dix-Hallpike) manoeuvres, normal vestibulo-ocular reflexes, and intact proprioception proximally and distally. An MRI of her head and cervical and thoracic spine and routine nerve conduction studies were normal. Somatosensory evoked potentials were performed due to absent ankle reflexes, which initially demonstrated delayed responses of both legs. Nevertheless, repeat testing revealed these to be of normal latency and symmetrical. Routine blood tests and standard serological tests for neuropathy were normal (including B12, folate, calcium, magnesium, bone profile, serum protein electrophoresis, HIV, syphilis, and Hepatitis B & C). Corrected visual acuity was normal. Vestibular function test including caloric test, vHIT, videonystagmography were also normal. She was diagnosed with primary FoF and began a tailored program of what we term “cognitive physical therapy,” combining cognitive behavioural therapy (CBT) techniques and physical rehabilitation (see below).

## Methods

### Assessment of Gait

Initial formal gait assessment was performed 8 months after symptom onset, then at 1 month and 3 months after initial assessment. The patient was assessed on her time to completion, number of steps taken and average stride length during a 6 m walk. This was compared with our (unpublished) database of healthy age-matched controls.

### Assessment of Mood and FoF

Measures of psychological variables and fear of falling were assessed using validated questionnaires. The Hospital Anxiety and Depression Scale (HADS), State and Trait Anxiety Inventory (STAI) and International Fall Efficacy Scale (FES-I) provided subjective measures of her anxiety and fear of falling. When answering the FES-I questionnaire, she provided answers for both aided (single walking aid) and unaided situations. Questionnaires were completed at initial assessment 8 months after symptom onset and 1 month later.

#### The Hospital Anxiety and Depression Scale

The HADS assesses anxiety (HADS-A) and depression (HADS-D) separately. Participants are asked to rate how they have felt in the last week (9).

#### The State and Trait Anxiety Inventory

The STAI consists of two subscales: the State Anxiety Scale (S-Anxiety) measuring the participant's current state of anxiety and the Trait Anxiety Scale (T-Anxiety) which measures the participant's anxiety feelings in general (10).

#### The International Falls Efficacy Scale

The short FES-I is a questionnaire that assesses participants' perception of their fall-related self-efficacy. Participants rank their concern about the possibility of falling in different scenarios (11).

### Existing Therapeutic Strategies

Traditional therapies for FoF are based around physical therapy, including muscle strengthening and increasing muscle flexibility or graded balance skills training (12) that are primarily aimed at avoidance of falls by reducing the impairments underlying the motor aspects of task performance such as reaction times or muscle strength (13).

FoF tends to increase with age, along with biomechanical sequalae such as weakness, joint and muscle changes, and alterations in balance performance. It is perhaps not surprising that traditional interventions reported in the literature are aimed at improving these requisites for performance of a motor behaviour as they have been shown to be related to falling risk (14, 15). Physical training is key to addressing risk of falls, and should be included when treating this group of patients, however, the impact of psychological sequalae in fear of falling has not been well-addressed in traditional physiotherapy approaches for falls (16).

Several authors have proposed the inclusion of task specific activity training within a multidimensional approach to fall prevention and rehabilitation, however this is typically described as a series of daily activity skills training—such as transfers, sit to stand or stair climbing. This may fall short of addressing the fear of falling within the context of the fear-inducing activity or location (17).

Cognitive Behavioural Therapy (CBT) is a psychotherapeutic intervention that targets maladaptive beliefs and re-direct behaviours toward positive strategies such as regular and safe exercise (18). FoF however may additionally generate maladaptive behaviours that alter posture and gait patterns (3, 19), that could themselves induce worsening balance and perpetuate FoF. Isolated CBT would not necessarily address these objective postural impairments.

### Cognitive Physical Therapy

The patient was offered a combination of “falls-oriented physical therapy” with aspects of CBT to provide a more holistic approach to treatment. Considering the patient's access to the health centre, the treatment programme consisted of three 30 min in-person sessions of “non-sedentary” therapy focussed on diverting attention on voluntary motor gait control through use of auditory cuing, and cognitive distractors during walking (that are used in functional gait disorders). The patient was encouraged to take longer strides in time to a self-generated beat or finger click, whilst using relaxation and realistic thinking strategies to explore, understand, and manage her underlying FoF-related anxiety. We focused on rationalisation of perceived postural instability to avoid “catastrophisation.” The patient was encouraged to practise the strategies learned during each session at home, first as a formal exercise session and later as part of her normal walking. Follow-up in person sessions occurred once every 4 weeks over a 3-month period and were delivered by a research audiologist trained in vestibular rehabilitation and CBT. The rehabilitation program and its delivery was based on evidence from systematic and meta-analysis reviews both CBT for fear of falling (5, 18) and vestibular rehabilitation for treatment of balance disorders (20), although there is no consensus for optimum frequency or treatment duration. The patient was not prescribed any additional pharmacological therapy.

## Results

### Gait Parameter Improvements

**Figure 1:**
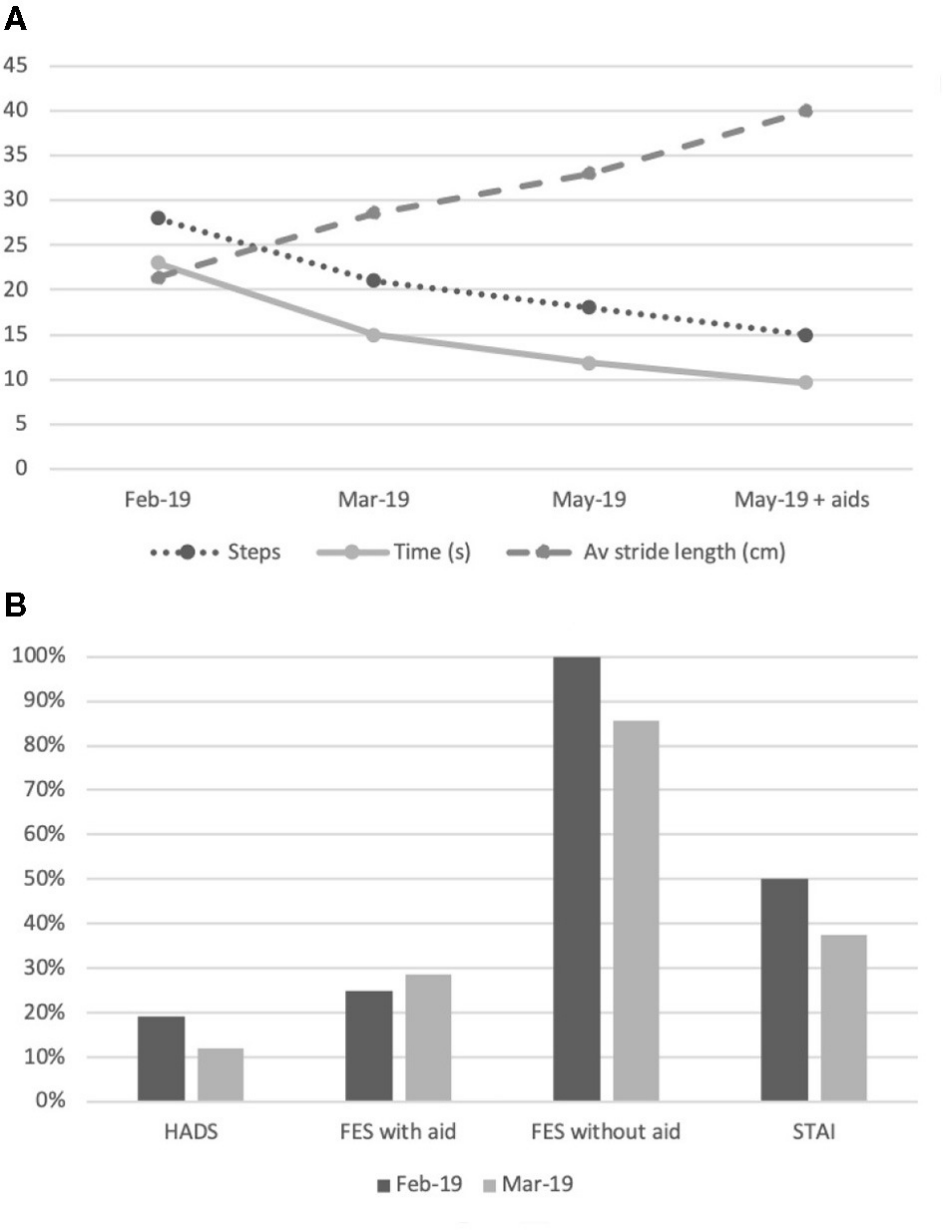
Summary of gait assessments and subjective measurements. **(A)** Changes in step count, time (seconds) and average stride length (cm) for a 6-metre walk. Data obtained at three time points: February 2019, March 2019, and May 2019. Further data provided in May 2019 for 6 m walk aided with a walking stick; **(B)** Subjective measures of anxiety and fear of falling with HADS, FES-I and STAI as a proportion of maximum possible scores at two time points: February 2019 and March 2019.

Formal gait assessment was performed 8 months after symptom onset; during a 6 m walk she took 28 steps in 23 s, with an average stride length of 21.4 cm (Figure 1A). Healthy controls of comparable age complete a 6 m walk with 10.4 steps in 6.9 s, with an average stride length of 57.7 cm (*n* = 15, unpublished data from out unit). During this session she was given advice on improving her gait and was instructed to adopt a rhythmic walk by marking the beat with her hands, and finger-clicks. Her progress was reviewed 1 month later when her 6 m walk time and steps were reduced by a 25 and 35%, respectively and the stride length increased by 34% (Figure 1A). She reported being able to walk longer distances unassisted, however, was still mostly reliant on her three-wheel walker. Qualitatively, her walk was more confident and less “shuffling.” During this second session she was advised to increase her stride length and walking speed and it was suggested that she replace the three-wheel walker with a single walking stick. Reassessment 2 months later again revealed a time reduction on the 6 m walk to 11.8 s, reduction in number of steps to 18, and increased stride length of 33 cm (Figure 1A; Supplementary File 2).

### Self-reported Measures

On initial assessment her total HADS score was 8, scoring 2 on HADS-A and 6 on HADS-D. Her STAI score was 40 and her FES was 7 with a walking aid and 28 unaided. This demonstrates low-grade anxiety, (which reduced even further in later evaluations), but a significant FoF when asked about her balance perception without the use of the walking aid.

On assessment 1 month later her HADS score was 5, scoring 1 on HADS-A and 4 on HADS-D. Her STAI score was 30 and her FES was 8 with a walking aid and 24 unaided. Despite the improvement in her gait and subjectively on her confidence, the reduction of her FoF when unaided was only marginal (14%, Figure 1B). Nevertheless, she reported subjective functional improvements with greater confidence when walking inside and also in the garden. After the second treatment session she was no longer using the three-wheeler walking aid for long distances and was completing shorter distances unaided. She was now performing tasks that she had previously avoided, a manifestation of improved balance confidence.

## Conclusion and Discussion

Whilst FoF is a common disorder in the elderly, it is likely to be under-recognised given the perceptual nature of the syndrome, lack of confirmatory tests and reluctance to seek medical help due to fear of stigmatisation (21). This case represents an unusually abrupt onset in an otherwise elderly healthy non-faller. The patient improved with a tailored “Cognitive Physical Therapy (CPT)” programme, embedding FoF within a cognitive neuroscience framework. CPT may have a role across a range of perceptual disturbances of gait and motor control in the elderly.

Causes of FoF are multifactorial and include prior falls, although this is not a prerequisite for developing FoF (22). Data from several case-control studies with FoF as an outcome measure identified other risk factors such as female gender, old age, dizziness, health status, depression, anxiety, poor mobility and poor self-perceived well-being (1, 11, 23–25). Traditional conceptualizations of FoF are based on fear avoidance, suggesting a cycle of FoF and subsequent avoidance of activity leading to muscle atrophy, worsening balance and gait and therefore falls (26). Hadjistavropoulos et al. adapted this model to incorporate multiple factors that are relevant in fear of falling including an individual's appraisal of their own ability to maintain balance which is affected by factors such as their self-assessed health status, fall risk factors and a history of previous falls (27). This more robust model does not however account for the acute onset of fear of falling–and subsequent improvement with CPT—that was apparent in our patient.

The acute onset of FoF demonstrated here suggests an abrupt shift in perceptual processing of self-stability. We have postulated that FoF may represent a heightened and permanent state of postural anxiety due to an internal awareness of altered balance function in elderly individuals, as evidenced by a negative correlation between the perception of stability and age (28). Interestingly, our patient demonstrated low-grade anxiety when assessed with HADS and STAI but significant FoF when assessed with FES-I. This suggests that her anxiety was directly related to falls and not symptomatic of a more generalised anxiety disorder. It also highlights the need for both objective and *subjective* measurements of FoF to ascertain the extent of the problem, construct a treatment protocol, and accurately track recovery.

**Figure 2:**
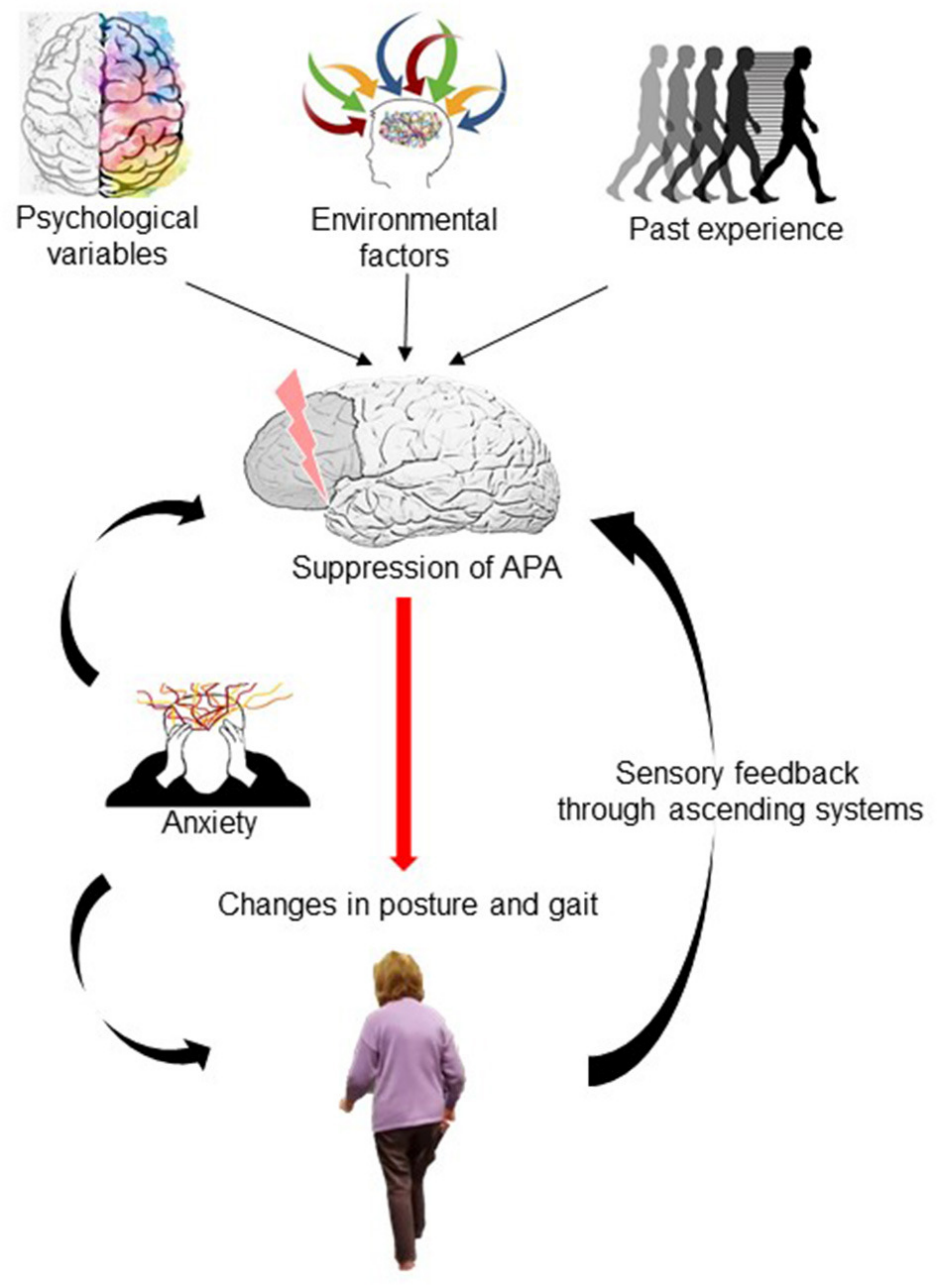
Diagrammatic schema for gait impairment in Fear of Falling. Past experiences (previous falls or near-falls) combine with psychological variables (state anxiety, bodily hypervigilance) and environmental factors (uneven or sloping surfaces) to alter prefrontal cortex activation leading to a suppression of anticipatory postural adjustment (APA) activity. This occurs in an attempt to increase balance performance and safety by reducing the size, speed and amplitude of such postural adjustments. This however becomes a less successful movement strategy, increasing risk of falls through stiffening of posture, and leads to heightened perception of falls risk through ascending sensory feedback, further cementing the suppression of APA. Postural anxiety increases postural stiffening and contributes to APA suppression.

Individuals with FoF may manifest heightened postural anxiety–irrespective of impaired balance function–through an awareness of the potential consequences of falling (e.g., fractures and facial injuries). Castro et al. investigated healthy subjects and demonstrated that whilst perception of instability is congruent with body sway across the ages, in older subjects a reduction in sway was not accompanied by a reduction in subjective measures of anxiety or instability (28). Thus, postural anxiety persists in the elderly in the absence of a postural threat. Considering the present case, the patient exhibited an immediate shift to a fearful response when exposed to a loss of stability, which translated for her into a significant increase in postural threat. The stimulus, which triggered the patient's symptoms, was equivalent for her in perceived magnitude to an actual fall, thus generating a central shift toward postural hypervigilance and anxiety and leading to persistent FoF. Such a shift to cortical-based control of posture appears to involve abnormal frontoparietal interactions (29). This is further supported by evidence implicating the prefrontal cortex in fear conditioning and extinction (30), decision confidence (31) and executive behaviour planning (32). Holtzer et al., assessed activation and efficiency of the prefrontal cortex in older participants with and without FoF during single-task and dual-task walking. They demonstrated greater activity in the prefrontal cortex when switching from single-task to dual-task walking in the FoF group. Since FoF subjects showed slower gait velocity, this finding suggests inefficient activation of the prefrontal cortex during dual-task walking. This inefficiency appears to be specific for attention-demanding locomotion as it was not present when completing isolated cognitive tasks (33). Thus, we postulate that inefficient prefrontal cortex activity may underpin the development of FoF in our patient (Figure 2). Such altered activity may in turn relate to an acute increase in postural anxiety.

Although fear responses have a primary psychological construct, FoF leads to measurable changes in balance behaviour and function. Adkin et al. (34) showed suppression in anticipatory postural adjustments (APA) activity in healthy normal individuals by changing the height of the task or the proximity to the edge of a raised platform (34). Naugle and colleagues (35) demonstrated direct effects on APA activity in gait initiation following exposure to positive or negatively arousing images, proposing direct modulation of motor circuitry via dopaminergic neurones via the basal ganglia (Naugle et al.). Suppression of APA activity, putatively mediated through prefrontal cortex top-down influences–may represent an attempt to increase balance performance and safety by reducing the size, speed and amplitude of such postural adjustments (Figure 2). Paradoxically, this becomes a less successful movement strategy, actually increasing risk of falls. This may account for the need for the treatment intervention to integrate task-based practise using graded environmental exposure/desensitisation, with cognitive coping strategies (including distraction/external cuing/coaching).

Gait assessment at presentation in our patient showed a reduced stride length, an increase in number of steps and time taken to complete a 6 m walk when compared with healthy controls. Such changes in gait correlate with anxiety in community-dwelling older adults (36). We demonstrated an improvement in all objective measures of gait with CPT, that were a behavioural consequence of the FoF. Perceptual measures of FoF were however less amenable to treatment over a short 1-month period, indicating perhaps the requirement for longer-term treatment. Moreover, FoF is a complex neuropsychological construct and perceptual or emotional variables may be more resistant to therapeutic interventions despite objective reduction in maladaptive gait strategies, and improvements in everyday function.

Traditional treatments for FoF focus on the use of physical therapy *or* CBT. Balance training, strength and resistance training and tai chi, have been used to treat FoF and have shown mild to moderate improvement in dynamic control and sensory integration (37). Specific and focussed guidelines to avoid falls in older people with a high fall risk have been developed by NICE and the Centres for Disease Control and Prevention, however, these are mainly focussed on home adaptations and muscular strengthening training, targeting fall avoidance specifically, but not FoF. Recommendations include the curtailment of possibly hazardous activities (13) in patients at risk of falls. Considering that our patient had restricted her movement to avoid falls due to a *perceived* but not objective fall risk, this recommendation may in fact be detrimental to recovery in patients with FoF with low falls risk.

The Strategies for Increasing Independence Confidence and Energy (STRIDE) study demonstrated a reduction in FoF with CBT delivered by healthcare assistants compared to usual care alone (referral to community exercise and home exercise) in the control group. However, they did not report a reduction in anxiety as measured by the HADS following CBT (5). Similarly, randomised controlled trials of cognitive intervention together with physical training reduces FoF and increases activity in healthy older adults with low levels of FoF (38). CBT-based multicomponent interventions for FoF are supported by meta-analysis data (39). Whether such benefits are also achievable in patients with higher burden of FoF has not been formally evaluated but our case suggests this may be possible. If we theorise that FoF is secondary to changes in the prefrontal cortex, then management programmes that specifically target prefrontal cortex efficiency, for example dual-task walking (33) should be incorporated into FoF interventions.

### Limitations

We acknowledge that this is a single case study and therefore the individualised therapy may not be generalisable to other elderly patients who present with FoF. Furthermore, there is no consensus regarding the optimum frequency and duration of physical and/or cognitive therapies for FoF, although we based our programme on evidence from systematic reviews and meta-analyses and were additionally guided by the time constraints and patient's geographical distance to the treatment centre.

In summary, whilst FoF often has an insidious onset, it may present abruptly in susceptible individuals and lead to postural anxiety and a shift to cortically-based postural control. CPT was helpful in improving objective gait parameters and reducing postural anxiety, perhaps by preventing (frontal lobe) cortical suppression of balance performance, in turn downregulating abnormal postural feedback to reduce postural anxiety.

## Data Availability Statement

The raw data supporting the conclusions of this article will be made available by the authors, without undue reservation.

## Ethics Statement

Ethical review and approval was not required for the study on human participants in accordance with the local legislation and institutional requirements. The patients/participants provided their written informed consent to participate in this study. Written informed consent was obtained from the individual(s) for the publication of any potentially identifiable images or data included in this article.

## Author Contributions

Material preparation, data collection and analysis were performed by PC, SV, and DK. All authors contributed to the study conception and design, commented on versions of the manuscript, read, and approved the final manuscript.

## Conflict of Interest

The authors declare that the research was conducted in the absence of any commercial or financial relationships that could be construed as a potential conflict of interest.

## Publisher's Note

All claims expressed in this article are solely those of the authors and do not necessarily represent those of their affiliated organizations, or those of the publisher, the editors and the reviewers. Any product that may be evaluated in this article, or claim that may be made by its manufacturer, is not guaranteed or endorsed by the publisher.
